# Effect of Mannan Oligosaccharides Extracts in Uropathogenic *Escherichia coli* Adhesion in Human Bladder Cells

**DOI:** 10.3390/pathogens12070885

**Published:** 2023-06-28

**Authors:** Margarida Faustino, Sara Silva, Eduardo M. Costa, Ana Margarida Pereira, Joana Odila Pereira, Ana Sofia Oliveira, Carlos M. H. Ferreira, Carla F. Pereira, Joana Durão, Manuela E. Pintado, Ana P. Carvalho

**Affiliations:** 1 CBQF–Centro de Biotecnologia e Química Fina–Laboratório Associado, Universidade Católica Portuguesa, Escola Superior de Biotecnologia, Rua Diogo Botelho 1327, 4169-005 Porto, Portugal; afaustino@ucp.pt (M.F.); jodila@ucp.pt (J.O.P.); assoliveira@ucp.pt (A.S.O.); chferreira@ucp.pt (C.M.H.F.); cpfpereira@ucp.pt (C.F.P.); jdurao@ucp.pt (J.D.); mpintado@ucp.pt (M.E.P.); apcarvalho@ucp.pt (A.P.C.); 2 Amyris Bio Products Portugal, Unipessoal Lda, Rua Diogo Botelho, 1327, 4169-005 Porto, Portugal

**Keywords:** uropathogenic *Escherichia coli* (UPEC), mannan oligosaccharides (MOS), adhesion, bladder cells, D-mannose, urinary tract infections (UTIs)

## Abstract

Urinary tract infections (UTIs) are a common public health problem, mainly caused by uropathogenic *Escherichia coli* (UPEC). Patients with chronic UTIs are usually treated with long-acting prophylactic antibiotics, which promotes the development of antibiotic-resistant UPEC strains and may complicate their long-term management. D-mannose and extracts rich in D-mannose such as mannan oligosaccharides (MOS; D-mannose oligomers) are promising alternatives to antibiotic prophylaxis due to their ability to inhibit bacterial adhesion to urothelial cells and, therefore, infection. This highlights the therapeutic potential and commercial value of using them as health supplements. Studies on the effect of MOS in UTIs are, however, scarce. Aiming to evaluate the potential benefits of using MOS extracts in UTIs prophylaxis, their ability to inhibit the adhesion of UPEC to urothelial cells and its mechanism of action were assessed. Additionally, the expression levels of the pro-inflammatory marker interleukin 6 (IL-6) were also evaluated. After characterizing their cytotoxic profiles, the preliminary results indicated that MOS extracts have potential to be used for the handling of UTIs and demonstrated that the mechanism through which they inhibit bacterial adhesion is through the competitive inhibition of FimH adhesins through the action of mannose, validated by a bacterial growth impact assessment.

## 1. Introduction

Urinary tract infections (UTIs) represent a severe public health problem and are caused by a range of pathogens, most commonly by *Escherichia coli* (*E. coli*), *Klebsiella pneumoniae* (*K. pneumoniae*), *Proteus mirabilis* (*P. mirabilis*), *Enterococcus faecalis* (*E. faecalis*) and *Staphylococcus saprophyticus* (*S. saprophyticus*) [1]. Globally, about 150 million people are affected by urinary tract infections each year, which are among the most common infectious diseases [2,3]. UTIs are the second leading cause of infections in the general population, predominantly in adult females, and are the leading cause of infection in hospital environments [4,5]. According to the European Association of Urology (EAU) Guidelines [6], they can be divided into uncomplicated (including lower UTIs (cystitis) and upper UTIs (pyelonephritis), typically affecting non-pregnant women, without any other health problem or anatomical/functional irregularities) and complicated (associated with individuals with increased probability of complications, namely pregnant women, men, or patients with other health-related problems such as renal anatomical/functional abnormalities, failure or transplantation, and other diseases, such as immunocompromised or diabetic individuals). In most cases, UTIs are caused by the pathogenic uropathogenic *E. coli* (UPEC), which is capable of migrating from the intestinal microbiota to the perianal region and, thereafter, to the urinary tract [7]. This bacterium is the most prevalent in symptomatic and/or asymptomatic infections, representing up to 75% to 95% of the reported UTIs [1,7,8], with the ability to colonize all parts of the urinary tract (including the urethra, ureters, kidney and bladder), thus causing acute, chronic, persistent, and recurrent infections [9,10]. Amidst the multitude of well-studied virulence factors, the ability of UPEC to adhere to host epithelial cells is the crucial step for the establishment and progression of infection [1,2,7,8]. This adhesion ability is mainly conferred by type 1 fimbriae D-mannose specific adhesin (FimH), located at the tip of UPEC’s type 1 fimbriae, which specifically binds to terminal epitopes of high mannosylated glycans conjugated to uroplakin 1a (UP1a), a receptor specifically expressed on the surface of urothelial cells [11,12,13,14,15,16].

D-mannose is widely used in the food, medicine, cosmetics, and food additive industries. Among its physiological health benefits, its positive effect on the immune system, diabetes mellitus (type 2), intestinal diseases, and urinary tract infections have been frequently reported [17,18]. D-mannose, a C-2 epimer of D-Glucose, and D-mannose analogues can prevent FimH-mediated bacterial adhesion through a competitive inhibition mechanism. This competition mechanism is based on the structural similarity between the D-mannose receptors and the urothelial mannosylated receptors exposed by the urinary tract epithelium [12,19,20,21,22]. When administered in sufficient amounts, D-mannose is rapidly absorbed and excreted through the urinary tract, where it will saturate UPEC’s FimH, thus blocking its binding and subsequent adhesion to the urothelium [23,24]. According to Scribano et al. [25], the D-mannose dosage range for UTI prevention determined in clinical trials is between 2 and 3 g per day and the normal urine volume is between 800 and 2000 mL/day. When administered orally, the absorption of D-mannose is rapid and detectable in plasma approximately 30 min after taking it, being then excreted thought the urinary tract. In addition to binding to bacterial fimbriae, D-mannose acts on the activation of the Tamm–Horsfall protein, which plays a key role in the body’s defense against UTIs [26,27].

In this work, the viability of using mannan oligosaccharides (MOS) as supplements for the management of UTIs was evaluated through the assessment of the ability of MOS extracts to inhibit the adhesion of UPEC to urothelial cells. Two MOS extracts, produced from yeast-purified mannans fractions by two different methodologies, were assessed and commercial D-mannose was used as the control. The expression levels of the pro-inflammatory interleukin 6 (IL-6) and the potential antimicrobial activity of all samples using a time–growth inhibition curve were also assessed.

## 2. Materials and Methods

### 2.1. Materials

MOS extracts were obtained from genetically modified spent yeast (*Saccharomyces cerevisiae*) from Amyris, Inc. (Emeryville, CA, USA) following a hydrothermal process at 110 °C for 3 h (MOS Parr) and an acidic process with phosphoric acid at 55 °C for 24 h (MOS H_3_PO_4_). Mannose content of these extracts was determined using gas-chromatography-flame ionization detection (GC-FID), as previously described [28]. D-mannose was purchased from Sigma-Aldrich, Munich, Germany.

### 2.2. Bacterial Strain and Cell Line

The well-characterized uropathogenic *E. coli* strain CFT073 (DSMZ, Braunschweig, Germany) was used in this study. Strain CFT073 was grown at 37 °C in Tryptic Soy Broth (TSB; Biokar Diagnostic, Beauvais, France) with 3 g/L of yeast extract (Sigma-Aldrich, Munich, Germany), from now on referred to only as TSB. The human bladder epithelial cell line 5637, HTB-9 was obtained from the American Type Culture Collection 5637 (ATCC-LGC, Milan, Italy) and routinely cultured in T75 flasks at 37 °C in a humidified atmosphere with 5% CO_2_ using Roswell Park Memorial Institute (RPMI 1640) medium (Invitrogen, Waltham, MA, USA) supplemented with 10% (*v*/*v*) heat-inactivated fetal bovine serum (FBS), with the addition of 1% (*v*/*v*) antibiotic 100x (Invitrogen, Waltham, MA, USA). Unless indicated otherwise, RPMI medium supplemented with FBS and without antibiotic will, from now one, be referred to only as RPMI.

### 2.3. Cytotoxicity Assay

The cytotoxic effect of the samples was assessed in HTB-9 cell line in conformity with the International Organization for Standardization (ISO) 10993-5 guideline [29], using the PrestoBlue™ Cell Viability Reagent (Thermo Fisher Scientific, Waltham, MA, USA) according to the instructions of the manufacturer. A stock solution of 30.00 mg/mL of each sample in RPMI medium with antibiotic was prepared, and D-mannose was used for comparation purposes. Different preparations were implemented due to the nature of their constituents and the stability of these compounds. For MOS H_3_PO_4_ and MOS Parr extracts were directly dissolved in RPMI medium with antibiotic and sterilized using a sterile syringe filter with a 0.22 µm pore size (Millipore, Billerica, MA, USA). Commercial D-mannose was dissolved in phosphate-buffered saline (PBS) solution at pH 7.4, to a final concentration 2-fold higher than the desired one. After being autoclaved for 20 min at 100 °C, a twofold dilution of the solution was prepared in antibiotic containing RPMI medium to achieve the final concentration of 30 mg/mL. For HTB-9 viability assay, cells in suspension were seeded at 1 × 10^4^ cells/well in a 96-well microtiter plate and maintained in culture for 24 h to form a semi-confluent monolayer. Following this incubation period, the cell culture medium was removed and replaced with the samples. Medium without the samples in each incubation period was used as positive cell viability control, whereas medium with a final concentration of 10% of DMSO was used as negative control for cytotoxicity. After an additional 24 h incubation period, PrestoBlue (PB) viable dye was added to the wells and changes in cell viability were detected using fluorescence spectroscopy. The fluorescence was read (λ excitation = 570 nm; λ emission = 610 nm) after incubation of 2 h.

### 2.4. Immunological Response without Stimulus

HTB-9 cells were seeded at a final concentration of 2.5 × 10^5^ cells/mL in a 24-well microtiter plate and immediately incubated at 37 °C with 5% CO_2_ in a humidified environment for 24 h. Following this incubation period, cells were washed twice with warm, sterile PBS to remove all the antibiotic-containing medium. HTB-9 cells were then exposed to the samples (MOS H_3_PO_4_, MOS Parr and D-mannose) diluted in RPMI medium at a non-cytotoxic mannose concentration. Medium without the samples was used as positive control in each incubation period and cells were incubated at 37 °C with 5% CO_2_ in a humidified environment for 1, 2 and 3 h. After that, the media were collected, transferred to microcentrifuge tubes, and kept at −20 °C until protein and IL-6 expression levels quantification (Section 2.6).

### 2.5. UPEC Adhesion Assays

#### 2.5.1. Simultaneous Exposure (Competition)

HTB-9 cells were seeded at a final concentration of 2.5 × 10^5^ cells/mL in a 24-well microtiter plate and immediately incubated at 37 °C with 5% CO_2_ in a humidified environment for 24 h. Following this incubation period, cells were washed twice with warm, sterile PBS to remove all the antibiotic-containing medium. UPEC previously grown in TBS were centrifuged (4700× *g*, 5 min, 4 °C), washed twice with PBS and resuspended in the same buffer at a multiplicity of infection (MOI) of 10 [25], while MOS H_3_PO_4_, MOS Parr and D-mannose samples were diluted in RPMI medium at a non-cytotoxic mannose concentration (2.5 mg/mL). HTB-9 cells in RPMI and infected with UPEC without MOS extracts were used as positive control in each incubation period. HTB-9 cells were then simultaneously exposed to the samples and to UPEC suspension in PBS and incubated for 2 h at the same incubation conditions mentioned above.

#### 2.5.2. Prophylaxis Assessment

Similar to the above-described study, HTB-9 cells were seeded at a final concentration of 2.5 × 10^5^ cells/mL in a 24-well microtiter plate and incubated at 37 °C with 5% CO_2_ in a humidified environment. After 24 h of incubation, medium was discarded, and cells were washed twice with warm, sterile PBS to remove any antibiotic traces. MOS H_3_PO_4_, MOS Parr and D-mannose were diluted in antibiotic-free RPMI medium at a mannose concentration of 2.5 mg/mL and added to the wells containing HTB-9 cells, which were then incubated at 37 °C with 5% CO_2_ in a humidified environment for 1, 2, and 3 h before infection of the urothelial cells with UPEC. After incubation, bacterial cells grown in TSB were centrifuged (4700× *g*, 5 min, 4 °C), washed twice with PBS, resuspended at MOI of 10 and added to the cells for 2 h (time for adhesion of the UPEC). HTB-9 cells in RPMI and infected with UPEC were used as positive control in each incubation period.

### 2.6. Total Viable Counts Determination

At the end of the comparison and prophylaxis assessments, media conditioned by the samples were transferred to a microcentrifuge tube and processed for protein and IL-6 expression level quantification, as described in Section 2.7. Cell monolayers were carefully washed twice with sterile, warm PBS to remove unbound bacteria and, after detachment with trypsin (TrypLE^TM^ Thermo Fisher Scientific, Waltham, MA, USA), cells were resuspended in PBS. After serial dilutions in 0.1% sterile peptone water (*w*/*v*), 100 µL of the cell suspensions was plated using the drop method [30] on Plate Count Agar (PCA) plates and incubated at 37 °C for 24 h before counting to determine the total viable counts (CFU/mL). Results are expressed as adhesion inhibition percentage (% Inhibition) calculated using Equation (1).

(1) $$\%\;\mathrm{Inhibition}\;=100-\left(\frac{\frac{CFU}{mL}of\;Sample}{\frac{CFU}{mL}of\;Control}\right)\times 100$$

### 2.7. Protein Determination and Interleukin Evaluation

Media conditioned by the samples and collected in microtubes at the end of each experiment were centrifuged (990× *g,* 20 min, 4 °C) and the supernatants were used for protein and IL-6 expression level quantification. Protein determination was performed in 96-well microtiter plates using the bicinchoninic acid (BCA) methodology, using the Pierce™ BCA Protein Assay Kit (Thermo Fisher Scientific, Waltham, MA, USA) according to the instructions of the manufacturer. IL-6 expression levels were assessed using enzyme-linked immunosorbent assay (ELISA) using the Human IL-6 Elisa Max™ Deluxe Kit (BioLegend, San Diego, CA, USA) according to the instructions of the manufacturer and results are presented in pg/µg of protein.

### 2.8. Antimicrobial Activity–Growth Inhibition Curves

The potential antimicrobial activity of H_3_PO_4_, MOS Parr and D-mannose was assessed using a time–growth inhibition curve [31]. A solution of each sample in the growth medium Mueller Hinton (MH) (Biokar Diagnostic, Beauvais, France) was prepared to a final concentration of non-cytotoxic mannose concentration (2.5 mg/mL). Mannose content of each sample was previously determined using GC-FID. MOS H_3_PO_4_ and MOS Parr extracts were diluted in MH medium and sterilized using a sterile 0.22 µm filter (Millipore, Billerica, MA, USA). D-mannose was dissolved in PBS solution to a final concentration 2-fold higher than the desired one and sterilized using the autoclave (Prohs, Porto, Portugal) at 100 °C for 20 min. After sterilization, D-mannose solution was twofold diluted in MH medium to achieve a final concentration, in mannose, of 2.5 mg/mL. The UPEC strain was used as a monoculture and before the assay was grown in Tryptic Soy Agar (TSA; Biokar Diagnostic, Beauvais, France) with 3 g/L of yeast extract (Sigma-Aldrich, Munich, Germany) at 37 °C for 24 h under aerobic conditions. Afterwards, one colony was picked, resuspended in 10 mL of MH broth, and grown at 37 °C for 24 h under aerobic conditions. The bacteria inoculum was adjusted to an optical density (OD) at 625 nm of 0.1–0.08 (cell density of 1 × 10^8^ cells/mL, corresponding to 1.5 × 10^7^ CFU/mL) and 10 times diluted to obtain the work inoculum. To evaluate bacterial growth inhibition, 980 µL of each sample was transferred to a sterile microtube and inoculated with 20 µL of the work UPEC inoculum. After mixing by vortexing, 200 µL of the suspensions was transferred to a 96-well microtiter plate (Nunc, Darmstadt, Germany) and the OD at 625 nm was assessed for a 24 h period at 37 °C (1 h intervals) using a microplate reader (Epoch, VT, USA) with the increase in OD values being considered a consequence of bacterial growth. A positive control was drawn using inoculated MH medium without antimicrobial agent and sterile MH medium was used as a negative control. Blanks of the samples were used to correct sample color OD interference.

### 2.9. Statistical Analysis

The normality of the samples was evaluated using Shapiro–Wilk’s Test. Two-way analysis of variance (ANOVA) with Tukey’s post-test and 95% confidence level was carried out with GraphPad Prism 7.04 software. Results are presented as mean values ± SD (standard deviation), and *p* < 0.05 was considered statistically significant. All experiments were performed in triplicate.

## 3. Results and Discussion

Before assessing the ability of the MOS extracts to inhibit the adhesion of UPEC to the urothelial cells and given their intended purpose as dietary supplements for UTI prevention, it was necessary to establish the cytotoxic profile of the samples against urothelial cells. D-mannose was used as the control and the immunomodulatory activity of the samples was also assessed.

### 3.1. Cytotoxicity

**Figure 1:**
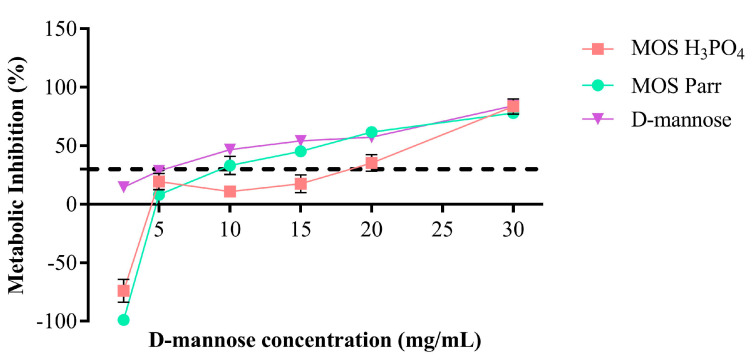
Cytotoxic effect on HTB-9 after 24 h of contact with the samples at different concentrations of D-mannose (mg/mL). Results are expressed as percentage of metabolic inhibition (%). Samples are only cytotoxic at concentrations of D-mannose above 5 mg/mL.

The cytotoxicity of the samples was assessed against HTB-9 cells by evaluating their impact upon cell metabolism using a viable dye (Figure 1) and taking into account the cytotoxicity threshold defined by ISO 10993-5 [29]—i.e., a sample is cytotoxic when a metabolic inhibition percentage above 30% is observed. Although a slight metabolic inhibition can be seen for mannose (14.79 ± 1%), according to the abovementioned standard, none of the samples was cytotoxic to HTB-9 cells at a mannose concentration of 2.5 mg/mL. In fact, the negative values observed for MOS Parr (−98.59 ± 0.34%) and MOS H_3_PO_4_ (−73.87 ± 9.91%) indicate an increase in cell metabolism when in the presence of these two extracts. When considering the total weight of extract and not just mannose, the 2.5 mg/mL of mannose translates into total extract concentrations of 4.17 mg/mL for MOS Parr and 4.10 mg/mL for MOS H_3_PO_4_.

### 3.2. Impact upon UPEC Growth

**Figure 2:**
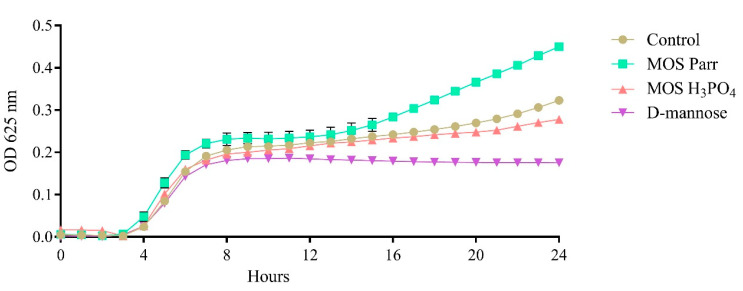
Effect upon the growth curves for UPEC exposed to the samples MOS extracts (Parr and H_3_PO_4_) and D-mannose.

To ensure that the inhibitions observed for the MOS extracts and D-mannose were a result of an inhibition of adhesion and not a consequence of their impact upon bacterial growth and survival, the samples’ influence on UPEC survival was studied by performing a growth inhibition assay, and the results are presented in Figure 2. As can be seen, the samples had no impact on the bacterial growth, with UPEC following the typical bacterial growth curve. Furthermore, it is possible to observe that, except for D-mannose, after entering the stationary phase, the number of viable bacteria in culture starts to increase again, albeit at a much slower pace than in the exponential phase.

Nevertheless, it was possible to demonstrate that none of the samples exerted any antimicrobial effect against UPEC.

### 3.3. Immunologic Response (1, 2 and 3 h Exposures to the Samples)

Enhancing the innate immune properties of the urothelium (bladder cells) represents an attractive approach for the prevention and treatment of UTIs, as the urothelium secretes and responds to chemokines and cytokines as an important component of its response to UTIs [32]. Produced by a myriad of cells such as T-cells, macrophages, fibroblasts, keratinocytes, glia cells, mesenchymal stem cells and endothelial cells, IL-6 is a pleiotropic cytokine that plays crucial roles in biological processes such as inflammation, immune response, and hematopoiesis [33,34]. IL-6 is expressed as a response to different stimulus molecules such as pathogen-associated molecular patterns (PAMPs) associated with pathogen infections or damage-associated molecular patterns (DAMPs) associated with damaged or dying cells due to trauma or burns [34,35], thus acting as inflammation biomarkers. Thus, MOS Parr, MOS H_3_PO_4_ and D-mannose samples were evaluated to determine their innate inflammatory profiles and immunomodulatory assays were performed, focusing on this cytokine, using the supernatants collected after HTB-9 cells’ exposure to the samples.

**Figure 3:**
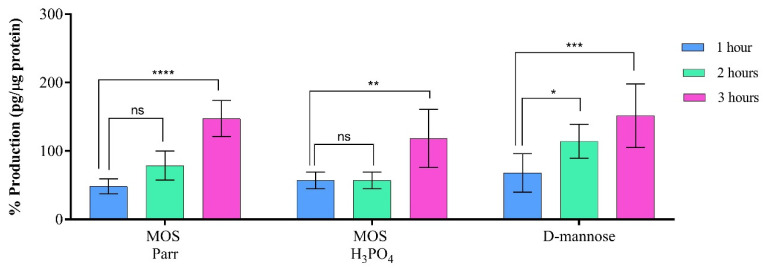
IL-6 values after HTB-9 cells were exposed to MOS H_3_PO_4_, MOS Parr and D-mannose samples. Results are expressed in relation to the control in each incubation period and bars represent means ± SD. * *p* < 0.05, ** *p* < 0.01, *** *p* < 0.001 and **** *p* < 0.0001 indicate statistically significant differences observed for the different incubations periods within samples (ns—non-significant).

The expression of IL-6 is depicted in Figure 3, revealing a tendency for increased IL-6 production by HTB-9 cells with longer incubation periods. Significant differences in cytokine expression were observed after 3 h of incubation with HTB-9 cells for MOS Parr (*p* < 0.0001), H_3_PO_4_ (*p* < 0.01), and D-mannose (*p* < 0.001). Except for specific time points, such as MOS Parr 1 h and 3 h, MOS H_3_PO_4_ 1 h, and D-mannose 3 h (*p* < 0.01), no significant differences (*p* > 0.05) were observed between the samples and their controls. Moreover, except for MOS H_3_PO_4_ after 2 h of incubation (*p* < 0.01), no significant differences in IL-6 production levels were found between the extracts and D-mannose (*p* > 0.05). MOS has been commercially available as a feed additive and, therefore, information reported regarding its immunomodulatory effect in humans is, to our knowledge, non-existent.

### 3.4. MOS Extracts Inhibitory Effect on UPEC Adhesion to HTB-9

**Figure 4:**
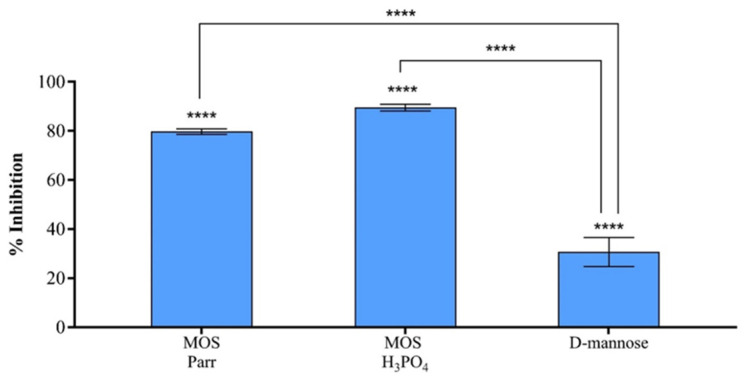
Percentage of adhesion inhibition of UPEC to HTB-9 after 2 h of simultaneous incubation with MOS H_3_PO_4_, MOS Parr and D-mannose Results are expressed in relation to the control and bars represent means ± SD. **** indicates statistically significant differences (*p* < 0.0001) between the MOS extracts and D-mannose, and between all samples and the control (HTB-9 cells in RPMI and infected with UPEC, used as reference for 100% survival).

To assess the adhesion inhibition of the MOS extracts, HTB-9 cells were simultaneously exposed to the samples and to a UPEC suspension. As can be seen in Figure 4, the adhesion inhibition values obtained for MOS H_3_PO_4_ (89.6 ± 1.4%), MOS Parr (79.8 ± 1.1%) and D-mannose (30.7 ± 5.9%) are significantly higher (*p* < 0.001) than those obtained for the control, demonstrating that all samples inhibited UPEC’s adhesion to HTB-9 cells. Furthermore, it was possible to observe that although no significant difference in bacterial adhesion was found between MOS H_3_PO_4_ and MOS Parr (*p* > 0.05), both extracts display a higher adhesion inhibition effectiveness than D-mannose (*p* < 0.001).

Due to their exploitation in animal supplementation as prebiotics, scientific reports on MOS have focused on their impact on the gastrointestinal microflora and immune system of farm animals [36,37,38,39,40]. Studies on the effect of MOS in UTIs are scarce [41], with most reports focusing on the benefit of using D-mannose as a therapeutic agent against UTIs [25,41,42,43,44,45]. One of the mechanisms through which UPEC is capable of adhering to cells is mediated by a bacterial ligand specific to D-mannose (FimH) located at the tip of type 1 pili anchored to UPEC’s outer membrane [1,11,46,47,48,49]. D-mannose thus inhibits bacterial binding to the mannosylated proteins (UP1a) expressed on the surface of urothelial cells [25,50], facilitating bacterial clearance though the urine flow.

### 3.5. Prophylactic Potential of MOS Extracts

**Figure 5:**
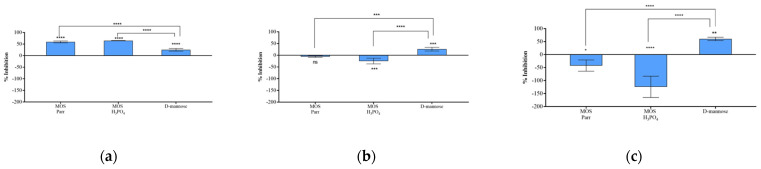
Percentage (%) of adhesion inhibition of UPEC to HTB-9 after 1 h (**a**), 2 h (**b**) and 3 h (**c**) of HTB-9 incubation with MOS H_3_PO_4_, MOS Parr and D-mannose. Bars represent means ± SD. * *p* < 0.05, ** *p* < 0.01, *** *p* < 0.001 and **** *p* < 0.0001 indicate statistically significant differences between the MOS extracts and D-mannose and between the different samples and the control (ns—non-significant). Results are expressed in relation to the control (HTB-9 cells in RPMI and infected with UPEC, used as reference for 100% survival).

To evaluate the prophylactic potential of MOS extract supplements, HTB-9 cells were incubated with the samples for 1, 2 and 3 h before infection with UPEC. Incubation periods were chosen considering that the majority of ingested mannose is filtered by the kidneys and excreted to the bladder via urine within 30 to 60 min [51] and that, although urinary frequency depends on a multitude of factors, it is commonly accepted that most people urinate over eight times in a 24 h period (up to 3 h intervals between voiding) [52,53]. The infection was allowed to occur for 2 h before the number of bacterial cells that were able to adhere to the urothelial cells was determined (Figure 5). The results demonstrated that, within the first hour of incubation (Figure 5a), all samples limited UPEC’s attachment to HTB-9 cells (*p* < 0.0001), with MOS Parr (59.3 ± 4.0%) and MOS H_3_PO_4_ (64.6 ± 0.9%) extracts inhibiting UPEC adhesion at significantly higher levels (*p* < 0.0001) than D-mannose (25.4 ± 5.6%). However, the inhibitory effect of the extracts significantly decreased (Figure 5b,c) after 2 h (*p* < 0.001 for MOS Parr and *p* < 0.0001 for H_3_PO_4_) and after 3 h (*p* < 0.0001 for both extracts) of incubation when compared with D-mannose, with this reduction being more substantial after 3 h. Indeed, the negative values observed for MOS Parr after 2 h (−5.19 ± 3.4%) and after 3 h of incubation (−42.3 ± 21.6%) and the negative values observed for MOS H_3_PO_4_ after 2 h (−24.6 ± 12.3%) and 3 h (−124.0 ± 41.0%) reveal that the extracts might promote bacterial adhesion to HTB-9 cells. A reasonable explanation for this observation may lay in the likely binding of cell proteins to the lipidic content of the MOS extracts, thus forming time-dependent protein–lipid complexes that would act as sort of “adhesion bridges” between the urothelium and the UPEC’s FimH binding sites saturated with mannose from the same MOS extracts. While not statistically significant (*p* > 0.05) for the MOS Parr extract after 2 h, this putative bacterial adhesion promotion can be slightly seen for MOS Parr after 3 h (*p* < 0.01) and is statistically evident for the MOS H_3_PO_4_ extracts after 2 h (*p* < 0.001) and after 3 h (*p* < 0.0001) of incubation with the urothelial cells. These results were in line with what has been previously reported by our group regarding the composition of MOS Parr and MOS H_3_PO_4_ extracts, when it was demonstrated that, although composed of the same percentage of protein, the latter possess a lower total sugar content, thus indicating a higher percentage, for instance, of lipids (among others) in MOS H_3_PO_4_, since MOS extracts are also composed of lipids [54]. Differently from what is observed with the MOS extracts, the inhibition efficacy of D-mannose was superior after 3 h of incubation with the HTB-9 cells (*p* < 0.05).

### 3.6. Cytokine Production in the Presence of UPEC

**Figure 6:**
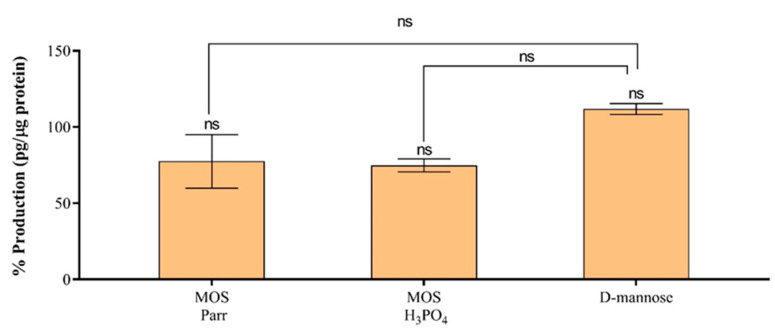
Quantification of IL-6 production levels by HTB-9 cells simultaneously exposed to UPEC, MOS extracts and D-mannose. No significant statistically differences (ns; *p* < 0.05) were found between MOS extracts and D-mannose nor between the different samples and the control. Bars represent means ± SD and results are expressed in relation to the control (HTB-9 cells in RPMI-medium-exposed UPEC, used as reference for 100% IL-6 expression).

**Figure 7:**
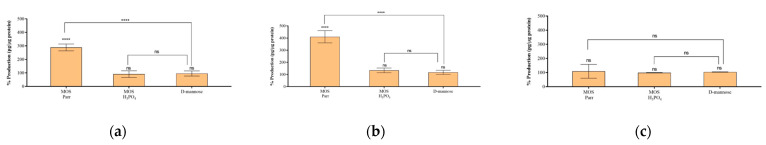
Quantification of IL-6 production levels by HTB-9 cells incubated with UPEC after exposition to MOS extracts and D-mannose for 1 h (**a**), 2 h (**b**) and 3 h (**c**). Bars represent means ± SD. **** indicates statistically significant differences (*p* < 0.0001) between MOS extracts and D.-mannose (ns—non-significant). Results are expressed in relation to the control (HTB-9 cells pretreated with RPMI medium before UPEC induced infection, used as reference for 100% survival at each incubation period).

In addition to providing a physical barrier against invading microorganisms, epithelium bladder cells also act as the first line of defense against urinary tract infections, with crucial roles such as pathogen recognition, the recruitment of phagocytes, the production of antimicrobial molecules and the release inflammatory mediators and cytokines [55]. IL-6 is rapidly excreted from urothelial cells after exposure to *Escherichia coli* [48,56,57]. Studies by De Man et al. [58], Wult et al. [59] and Schiling et al. [60] have implicated bacterial determinants such as lipopolysaccharides (LPS) and P-fimbriae in the urothelial production of IL-6. In the bladder urothelium, LPS interacts with Toll-like receptor (TLR) 4 and triggers an intracellular signaling cascade, leading to IL-6 secretion [48,56,60]. Thus, and considering that the bladder is commonly a sterile site, UTI generates a local powerful inflammatory response, with neutrophils, IL-6 and IL-8 being found in the urine of affected individuals [61,62]. IL-6 expression levels can be an indicator of the severity of clinical UTIs [9,63]. Indeed, children with pyelonephritis and renal scarring have been reported to exhibit higher IL-6 levels in urine and serum than those of children with cystitis [64,65,66,67]. To assess the effect of the samples in the immune response of bladder cells, the expression levels of the pro-inflammatory IL-6 were assessed in HTB-9 cells simultaneously exposed to the samples and subjected to UPEC infection at an MOI of 10 (Figure 6). This effect was also evaluated in HTB-9 cells infected with UPEC after pre-treatment with the samples for 1 h, 2 h and 3 h (Figure 7).

The determination of the expression levels of IL-6 on the supernatants of HTB-9 cell cultures exposed simultaneously to the samples and to UPEC (Figure 6) revealed no significant alteration (*p* > 0.05) in cytokine secretion when compared to cells in RPMI infected with UPEC (control). Moreover, the expression levels of IL-6 obtained for cells exposed to both MOS extracts and those obtained for cells exposed to D-mannose were not statistically different (*p* > 0.05). Given these results, it was reasonable to conclude that MOS extracts and D-mannose were not immunomodulatory and that only bacteria appeared to be contributing to the immune response of the urothelium cells observed. This conclusion was further supported by the results attained with HTB-9 cells pre-treated with the samples (Figure 7), where no synergetic effect was observed (*p* > 0.05) between the samples and the bacteria, except for cells pre-treated with MOS Parr for 1 h and 2 h (*p* < 0.0001), for which it was possible to see an exacerbation of the pro-inflammatory response (Figure 7a,b). The observed behavior of the initial response followed by a subsequent decrease in activity could potentially be attributed to the breakdown of a component that can be metabolized by the cells or the formation of complexes with certain components in the medium, leading to a loss of effectiveness. This hypothesis suggests that the component’s activity may be compromised over time, resulting in the observed decrease in response.

Overall, the results reported here highlight the potential use of MOS extracts as a dietary supplement for handling UTIs. These promising results must, however, be validated with samples submitted to an in vitro simulation of the gastrointestinal tract (GIT) system, since it is known that, to reach their destination, bladder-targeted food supplements must, firstly, go through a digestion process and mainly through an absorption process that can alter the structure of the molecules that compose them and, consequently, their activity with consequences in the amount absorbed that may not be sufficient to produce the desired effect. GIT simulation could also show that MOS could be effective for prophylaxis against recurrent UTIs, since, among other events, it is possible that digestive enzymes, namely lipases, will hydrolyze the lipids that are part of the constitution of MOS extracts and that may be responsible for the bacterial adhesion promotion by binding to cell proteins. Furthermore, the processes of digestion will have to be taken into account when evaluating the possible ways of administering MOS supplements, such as gummies, water-soluble powders and gastro-resistant capsules.

## 4. Conclusions

In this study, the feasibility of using MOS extracts to prevent UTIs was assessed and although their prophylactic capacity can be hindered by some unexpected interaction with the cells, they have potential as a dietary supplement used to address UTIs, due to its outstanding competitive inhibition of UPEC adherence to urothelial cells. Indeed, the competitive assay demonstrated that the effectiveness of bacterial adherence inhibition to bladder cells of the MOS was 2- to 3-fold higher than the one of D-mannose. Moreover, MOS extracts showed no significant immunomodulatory effect or evoke a cytotoxic response up to 2.5 mg/mL of mannose concentration. Furthermore, GIT simulation studies may potentially disclose a further potential of MOS for prophylaxis against recurrent UTIs.

## Data Availability

The data presented in this study are available within the article.

## References

[1] Flores-Mireles A.L., Walker J.N., Caparon M., Hultgren S.J. (2015). Urinary Tract Infections: Epidemiology, Mechanisms of Infection and Treatment Options. Nat. Rev. Microbiol..

[2] Terlizzi M.E., Gribaudo G., Maffei M.E. (2017). UroPathogenic *Escherichia coli* (UPEC) Infections: Virulence Factors, Bladder Responses, Antibiotic, and Non-Antibiotic Antimicrobial Strategies. Front. Microbiol..

[3] Kalas V., Hibbing M.E., Maddirala A.R., Chugani R., Pinkner J.S., Mydock-McGrane L.K., Conover M.S., Janetka J.W., Hultgren S.J. (2018). Structure-Based Discovery of Glycomimetic FmlH Ligands as Inhibitors of Bacterial Adhesion during Urinary Tract Infection. Proc. Natl. Acad. Sci. USA.

[4] Magliano E., Grazioli V., Deflorio L., Leuci A.I., Mattina R., Romano P., Cocuzza C.E. (2012). Gender and Age-Dependent Etiology of Community-Acquired Urinary Tract Infections. Sci. World J..

[5] Vyas S., Varshney D., Sharma P., Juyal R., Nautiyal V., Shrotriya V. (2015). An Overview of the Predictors of Symptomatic Urinary Tract Infection among Nursing Students. Ann. Med. Health Sci. Res..

[6] Bonkat G., Bartoletti R., Bruyère F., Cai T., Geerlings S.E., Köyes B., Kranz J., Schubert S., Pilatz A., Veeratterapillay R. (2023). EAU Guidelines, Proceedings of the EAU Annual Congress, Milan, Italy, 10–13 March 2023.

[7] McLellan L.K., Hunstad D.A. (2016). Urinary Tract Infection: Pathogenesis and Outlook. Trends Mol. Med..

[8] Forsyth V.S., Armbruster C.E., Smith S.N., Pirani A., Springman A.C., Walters M.S., Nielubowicz G.R., Himpsl S.D., Snitkin E.S., Mobley H.L.T. (2018). Rapid Growth of Uropathogenic *Escherichia coli* during Human Urinary Tract Infection. mBio.

[9] Hannan T.J., Mysorekar I.U., Hung C.S., Isaacson-Schmid M.L., Hultgren S.J. (2010). Early Severe Inflammatory Responses to Uropathogenic *E. coli* Predispose to Chronic and Recurrent Urinary Tract Infection. PLOS Pathog..

[10] Schwartz D.J., Chen S.L., Hultgren S.J., Seed P.C. (2011). Population Dynamics and Niche Distribution of Uropathogenic Escherichia Coli during Acute and Chronic Urinary Tract Infection. Infect. Immun..

[11] Ribić R., Meštrović T., Neuberg M., Kozina G. (2018). Effective Anti-Adhesives of Uropathogenic *Escherichia coli*. Acta Pharm..

[12] Mydock-Mcgrane L.K., Cusumano Z.T., Janetka J.W. (2016). Mannose-Derived FimH Antagonists: A Promising Anti-Virulence Therapeutic Strategy for Urinary Tract Infections and Crohn’s Disease. Expert Opin. Ther. Pat..

[13] Mickiewicz K.M., Kawai Y., Drage L., Gomes M.C., Davison F., Pickard R., Hall J., Mostowy S., Aldridge P.D., Errington J. (2019). Possible Role of L-Form Switching in Recurrent Urinary Tract Infection. Nat. Commun..

[14] Anderson G.G., Palermo J.J., Schilling J.D., Roth R., Heuser J., Hultgren S.J. (2003). Intracellular Bacterial Biofilm-like Pods in Urinary Tract Infections. Science.

[15] Snyder J.A., Lloyd A.L., Lockatell C.V., Johnson D.E., Mobley H.L.T. (2006). Role of Phase Variation of Type 1 Fimbriae in a Uropathogenic Escherichia Coli Cystitis Isolate during Urinary Tract Infection. Infect. Immun..

[16] Wu X.R., Sun T.T., Medina J.J. (1996). In Vitro Binding of Type 1-Fimbriated *Escherichia coli* to Uroplakins Ia and Ib: Relation to Urinary Tract Infections. Proc. Natl. Acad. Sci. USA.

[17] Wei Z., Huang L., Cui L., Zhu X. (2020). Mannose: Good Player and Assister in Pharmacotherapy. Biomed. Pharmacother..

[18] Wu H., Zhang W., Mu W. (2019). Recent Studies on the Biological Production of D-Mannose. Appl. Microbiol. Biotechnol..

[19] Cusumano C.K., Pinkner J.S., Han Z., Greene S.E., Ford B.A., Crowley J.R., Henderson J.P., Janetka J.W., Hultgren S.J. (2011). Treatment and Prevention of Urinary Tract Infection with Orally Active FimH Inhibitors. Sci. Transl. Med..

[20] Firon N., Ofek I., Sharon N. (1982). Interaction of Mannose-Containing Oligosaccharides with the Fimbrial Lectin of *Escherichia coli*. Biochem. Biophys. Res. Commun..

[21] Klein T., Abgottspon D., Wittwer M., Rabbani S., Herold J., Jiang X., Kleeb S., Lüthi C., Scharenberg M., Bezençon J. (2010). FimH Antagonists for the Oral Treatment of Urinary Tract Infections: From Design and Synthesis to in vitro and in vivo Evaluation. J. Med. Chem..

[22] Ofek I., Mirelman D., Sharon N. (1977). Adherence of *Escherichia coli* to Human Mucosal Cells Mediated by Mannose Receptors. Nature.

[23] O’Brien V.P., Hannan T.J., Nielsen H.V., Hultgren S.J. (2016). Drug and Vaccine Development for the Treatment and Prevention of Urinary Tract Infections. Microbiol. Spectr..

[24] Zacchè M.M., Giarenis I. (2016). Therapies in Early Development for the Treatment of Urinary Tract Inflammation. Expert Opin. Investig. Drugs.

[25] Scribano D., Sarshar M., Prezioso C., Lucarelli M., Angeloni A., Zagaglia C., Palamara A.T., Ambrosi C. (2020). D-Mannose Treatment Neither Affects Uropathogenic *Escherichia coli* Properties nor Induces Stable Fimh Modifications. Molecules.

[26] Pigrau C., Escolà-Vergé L. (2020). Recurrent Urinary Tract Infections: From Pathogenesis to Prevention. Med. Clin..

[27] Serafini-Cessi F., Monti A., Cavallone D. (2005). N-Glycans Carried by Tamm-Horsfall Glycoprotein Have a Crucial Role in the Defense against Urinary Tract Diseases. Glycoconj. J..

[28] Faustino M., Durão J., Pereira C.F., Oliveira A.S., Pereira J.O., Pereira A.M., Ferreira C., Pintado M.E., Carvalho A.P. (2022). Comparative Analysis of Mannans Extraction Processes from Spent Yeast *Saccharomyces cerevisiae*. Foods.

[29] (2009). Biological Evaluation of Medical Devices. Part 5: Tests for in vitro Cytotoxicity.

[30] Miles A.A., Misra S.S., Irwin J.O. (1938). The Estimation of the Bactericidal Power of the Blood. J. Hyg..

[31] (2012). CLSI Methods for Dilution Antimicriobial Susceptibility Tests for Bacteria That Grow Aerobically.

[32] Svanborg C., Godaly G., Hedlund M. (1999). Cytokine Responses during Mucosal Infections: Role in Disease Pathogenesis and Host Defence. Curr. Opin. Microbiol..

[33] Kishimoto T. (2005). Interleukin-6: From Basic Science to Medicine—40 Years in Immunology. Annu. Rev. Immunol..

[34] Tanaka T., Narazaki M., Kishimoto T. (2014). IL-6 in Inflammation, Immunity, and Disease. Cold Spring Harb. Perspect. Biol..

[35] Moriyama K., Nishida O. (2021). Targeting Cytokines, Pathogen-Associated Molecular Patterns, and Damage-Associated Molecular Patterns in Sepsis via Blood Purification. Int. J. Mol. Sci..

[36] Park J., Jung S., Carey J.B. (2019). Effects of a Commercial Mannan-Oligosaccharide Product on Growth Performance, Intestinal Histomorphology, and Amino Acid Digestibility in White Pekin Ducks. J. Appl. Poult. Res..

[37] Zheng C., Zhou J., Zeng Y., Liu T. (2021). Effects of Mannan Oligosaccharides on Growth Performance, Nutrient Digestibility, Ruminal Fermentation and Hematological Parameters in Sheep. PeerJ.

[38] Hooge D.M., Sims M.D., Sefton A.E., Connolly A., Spring P. (2003). Effect of Dietary Mannan Oligosaccharide, With or Without Bacitracin or Virginiamycin, on Live Performance of Broiler Chickens at Relatively High Stocking Density on New Litter. J. Appl. Poult. Res..

[39] Agazzi A., Perricone V., Omodei Zorini F., Sandrini S., Mariani E., Jiang X.-R., Ferrari A., Crestani M., Nguyen T.X., Bontempo V. (2020). Dietary Mannan Oligosaccharides Modulate Gut Inflammatory Response and Improve Duodenal Villi Height in Post-Weaning Piglets Improving Feed Efficiency. Animals.

[40] Halas V., Nochta I. (2012). Mannan Oligosaccharides in Nursery Pig Nutrition and Their Potential Mode of Action. Animals.

[41] Brown C., Katz R., McCulloch M. (2012). Yeast Mannan Oligosaccharide Dietary Supplement in the Treatment of Chronically Acute Urinary Tract Infections: A Case Series. UroToday Int. J..

[42] Kim D.Y., Lee J.C. (2017). Adherence Assay of Uropathogenic *Escherichia coli* in vivo and *in vitro*. Urogenit. Tract Infect..

[43] Porru D., Parmigiani A., Tinelli C., Barletta D., Choussos D., Di Franco C., Bobbi V., Bassi S., Miller O., Gardella B. (2014). Oral D-Mannose in Recurrent Urinary Tract Infections in Women: A Pilot Study. J. Clin. Urol..

[44] Ruggieri M.R., Hanno P.M., Levin R.M. (1985). Mannose Inhibition of Escherichia Coli Adherence to Urinary Bladder Epithelium: Comparison with Yeast Agglutination. Urol. Res..

[45] Taleb N.S. (2018). A Study of Therapeutic Effect of D-Mannose on Urinary Infection Caused by *Escherichia coli*. MOJ Proteom. Bioinforma..

[46] Crépin S., Houle S., Charbonneau M.È., Mourez M., Harel J., Dozois C.M. (2012). Decreased Expression of Type 1 Fimbriae by a Pst Mutant of Uropathogenic *Escherichia coli* Reduces Urinary Tract Infection. Infect. Immun..

[47] Schwan W.R., Beck M.T., Hung C.S., Hultgren S.J. (2018). Differential Regulation of *Escherichia coli* Fim Genes Following Binding to Mannose Receptors. J. Pathog..

[48] Samuelsson P., Hang L., Wullt B., Irjala H., Svanborg C. (2004). Toll-like Receptor 4 Expression and Cytokine Responses in the Human Urinary Tract Mucosa. Infect. Immun..

[49] Flores-Mireles A.L., Walker J.N., Bauman T.M., Potretzke A.M., Schreiber H.L., Park A.M., Pinkner J.S., Caparon M.G., Hultgren S.J., Desai A. (2016). Fibrinogen Release and Deposition on Urinary Catheters Placed during Urological Procedures. J. Urol..

[50] Sarshar M., Behzadi P., Ambrosi C., Zagaglia C., Palamara A.T., Scribano D. (2020). FimH and Anti-Adhesive Therapeutics: A Disarming Strategy against Uropathogens. Antibiotics.

[51] Scaglione F., Musazzi U.M., Minghetti P. (2021). Considerations on D-Mannose Mechanism of Action and Consequent Classification of Marketed Healthcare Products. Front. Pharmacol..

[52] McAchran S., Rackley R., Vasavada S. (2009). Neuromodulation for Voiding Dysfunction. Neuromodulation.

[53] Lukacz E.S., Whitcomb E.L., Lawrence J.M., Nager C.W., Luber K.M. (2009). Urinary Frequency in Community-Dwelling Women: What Is Normal?. Am. J. Obstet. Gynecol..

[54] Faustino M., Durão J., Pereira C.F., Pintado M.E., Carvalho A.P. (2021). Mannans and Mannan Oligosaccharides (MOS) from *Saccharomyces cerevisiae*—A Sustainable Source of Functional Ingredients. Carbohydr. Polym..

[55] Spencer J.D., Schwaderer A.L., Becknell B., Watson J., Hains D.S. (2014). The Innate Immune Response during Urinary Tract Infection and Pyelonephritis. Pediatr. Nephrol..

[56] Schilling J.D., Martin S.M., Hunstad D.A., Patel K.P., Mulvey M.A., Justice S.S., Lorenz R.G., Hultgren S.J. (2003). CD14- and Toll-like Receptor-Dependent Activation of Bladder Epithelial Cells by Lipopolysaccharide and Type 1 Piliated *Escherichia coli*. Infect. Immun..

[57] Hedges S., Agace W., Svensson M., Sjogren A.C., Ceska M., Svanborg C. (1994). Uroepithelial Cells Are Part of a Mucosal Cytokine Network. Infect. Immun..

[58] De Man P., Van Kooten C., Aarden L., Engberg I., Linder H., Svanborg Eden C. (1989). Interleukin-6 Induced at Mucosal Surfaces by Gram-Negative Bacterial Infection. Infect. Immun..

[59] Wullt B., Bergsten G., Connell H., Röllano P., Gebratsedik N., Hang L., Svanborg C. (2001). P-Fimbriae Trigger Mucosal Responses to *Escherichia coli* in the Human Urinary Tract. Cell. Microbiol..

[60] Schilling J.D., Mulvey M.A., Vincent C.D., Lorenz R.G., Hultgren S.J. (2001). Bacterial Invasion Augments Epithelial Cytokine Responses to *Escherichia coli* Through a Lipopolysaccharide-Dependent Mechanism. J. Immunol..

[61] Graham J.C., Galloway A. (2001). ACP Best Practice No 167: The Laboratory Diagnosis of Urinary Tract Infection. J. Clin. Pathol..

[62] Jantausch B.A., O’Donnell R., Wiedermann B.L. (2000). Urinary Interleukin-6 and Interleukin-8 in Children with Urinary Tract Infection. Pediatr. Nephrol..

[63] Schwartz D.J., Conover M.S., Hannan T.J., Hultgren S.J. (2015). Uropathogenic *Escherichia coli* Superinfection Enhances the Severity of Mouse Bladder Infection. PLOS Pathog..

[64] Tramma D., Hatzistylianou M., Gerasimou G., Lafazanis V. (2012). Interleukin-6 and Interleukin-8 Levels in the Urine of Children with Renal Scarring. Pediatr. Nephrol..

[65] Sheu J.-N., Chen M.-C., Chen S.-M., Chen S.-L., Chiou S.-Y., Lue K.-H. (2009). Relationship between Serum and Urine Interleukin-6 Elevations and Renal Scarring in Children with Acute Pyelonephritis. Scand. J. Urol. Nephrol..

[66] Sheu J.N., Chen M.C., Lue K.H., Cheng S.L., Lee I.C., Chen S.M., Tsay G.J. (2006). Serum and Urine Levels of Interleukin-6 and Interleukin-8 in Children with Acute Pyelonephritis. Cytokine.

[67] Rodríguez L.M., Robles B., Marugán J.M., Suárez Á., Santos F. (2008). Urinary Interleukin-6 Is Useful in Distinguishing between Upper and Lower Urinary Tract Infections. Pediatr. Nephrol..

